# Exosomes in melanoma: a role in tumor progression, metastasis and impaired immune system activity

**DOI:** 10.18632/oncotarget.24846

**Published:** 2018-04-17

**Authors:** Marco Tucci, Francesco Mannavola, Anna Passarelli, Luigia Stefania Stucci, Mauro Cives, Franco Silvestris

**Affiliations:** ^1^ Department of Biomedical Sciences and Human Oncology, University of Bari Aldo Moro, Bari, Italy

**Keywords:** exosomes, melanoma, immune system

## Abstract

Exosomes (Exo) are small vesicles produced by melanoma cells and the accessory cells of the tumor microenvironment. They emerge via both classical and direct pathways and actively participate in tumor colonisation of distant tissues. The proteins, nucleic acids, cytokines and growth factors engulfed by Exo are transferred to recipient cells, where they drive numerous functions required for the tumor escape from immune system control and tumor progression. By positively or negatively modulating immune cell properties, Exo provoke immune suppression and, in turn, defective dendritic cell (DC) functions. Together, these effects limit the cytotoxicity of T-cells and expand both T-regulatory and myeloid-derived suppressor populations. They also hinder perforin and granzyme production by natural killer cells. Finally, Exo also control the organotropism of melanoma cells. The distinct phenotypic properties of Exo can be exploited both for diagnostic purposes and in the early identification of melanoma patients likely to respond to immunotherapy. The potential therapeutic application of Exo derived from DCs has been demonstrated in vaccination trials, which showed an increase in anti-melanoma activity with respect to circulating tumor cells. However, additional studies are required before Exo can be effectively used in diagnostic and therapeutic applications in melanoma.

## INTRODUCTION

Cutaneous melanoma is a very aggressive cancer whose incidence has rapidly increased worldwide. The prognosis is generally poor given the propensity of melanoma cells to spread to distant sites while evading immune system control [1]. The relevant events that favour the immune escape of melanoma cells include the variable antigenic profile of the tumor cells, the enrolment of suppressor cells, the release of soluble factors within the microenvironment and the propagation of signals driven by melanoma microRNAs (miRNAs) [2, 3]. Both tumor expansion and the development of metastasis are regulated by molecular mechanisms that reflect the continuous cross-talk between cancer cells and the surrounding stromal components that critically accelerates or restrains cancer progression [4]. In addition to direct cell-to-cell contact and the indirect interplay of melanoma cells with the stroma through the release of soluble factors, recent studies have provided evidence of other mechanisms of communication, including the secretion by both malignant cells and immune cells of small vesicles that have engulfed the cellular proteins, DNAs and RNAs actively involved in melanoma progression and metastasis propagation [5].

Eukaryotic and prokaryotic cells continuously secrete spherical vesicles composed of a phospholipid bilayer with variable size and of cytosolic origin [6]. These vesicles include microparticles, apoptotic bodies and exosomes (Exo) [7]. Exosomes are produced by normal as well as malignant cell populations and drive a number of specialised functions implicated in intercellular signalling, protein cargo transport, proliferation and cancer development [8]. They are small vesicles formed by the invagination of the late endosome membrane and thus enriched in cytoplasmic components such as the extracellular domains of different receptors [9].

Exosomes regulate many cellular functions implicated in the proliferation of melanoma cells already primed to invade distant tissues by activating the epithelial-mesenchymal transition (EMT) and inducing pre-metastatic niche formation [10]. They also control the intracellular signals required for the degradation of the extracellular matrix (ECM) by metalloproteases (MMPs) activated downstream integrins, epidermal growth factor and Notch receptors [11, 12].

By regulating the differentiation and maturation of dendritic cells (DCs) and their antigen-processing ability, Exo regulate immune cell activity and counteract anti-melanoma immune processes [13] while also modulate apoptosis and the survival of CD4^+^ and CD8^+^ effector T-cells (Teffs), regulatory T-cells (Treg) and myeloid-derived suppressor cells (MDSCs) [14–17]. Cytokine production in the vicinity of tumor cells and the inhibition of the cytotoxicity of natural killer (NK) cells are also mediated by Exo [18]. However, their role in cancer progression can also be exploited in therapeutic applications since Exo usually carry tumor-derived antigens processed by DCs that elicit a functional immune response. In fact, the potential therapeutic application of Exo has been evaluated in experimental clinical trials of Exo-based vaccination [19].

In addition, antigen expression by Exo isolated from glioblastoma [20], ovarian [21] and prostate cancer was reported to be of diagnostic value [22]. However, in melanoma a reliable diagnostic and prognostic significance of Exo has yet to be demonstrated [23]. Our group recently demonstrated that the high levels of CD28 and PD-1 exposed by Exo correlate with the therapeutic response to anti-CTLA4 immunotherapy in metastatic melanoma, whereas those from DCs reflect the restoration of immune system activity against melanoma cells [24]. Here, we review recent studies on the origin, biological functions, diagnostic and therapeutic implications of Exo in melanoma.

## CLASSIFICATION, BIOLOGY AND PATHWAYS OF EXOSOME FORMATION

Extracellular vesicles are primarily classified as 1) Exo, 2) microvesicles, 3) membrane particles and 4) apoptotic bodies [25, 26]. Exosome appear as 30- to 120-nm vesicles with a cup-shaped morphology that is useful for their distinction from other, similarly sized particles [27]. Exo also characteristically have a typical protein and lipid composition derived from endosomal compartments and including tetraspanins (CD9, CD63, CD81 and CD82), proteins related to multivesicular body biogenesis (Alix and Tsg101), heat shock proteins (Hsp90 and Hsc70), transport proteins (GTPases, annexins and flotillin) and integrins, while large amounts of cholesterol, sphingomyelin and ceramide surround their lipid bilayer [9, 28, 29]. The outer surface of Exo is enriched in saccharide groups, such as mannose, sialic acid and glycans, whereas phosphatidylethanolamine is mostly found between the two leaflets of the bilayer [28].

**Figure 1:**
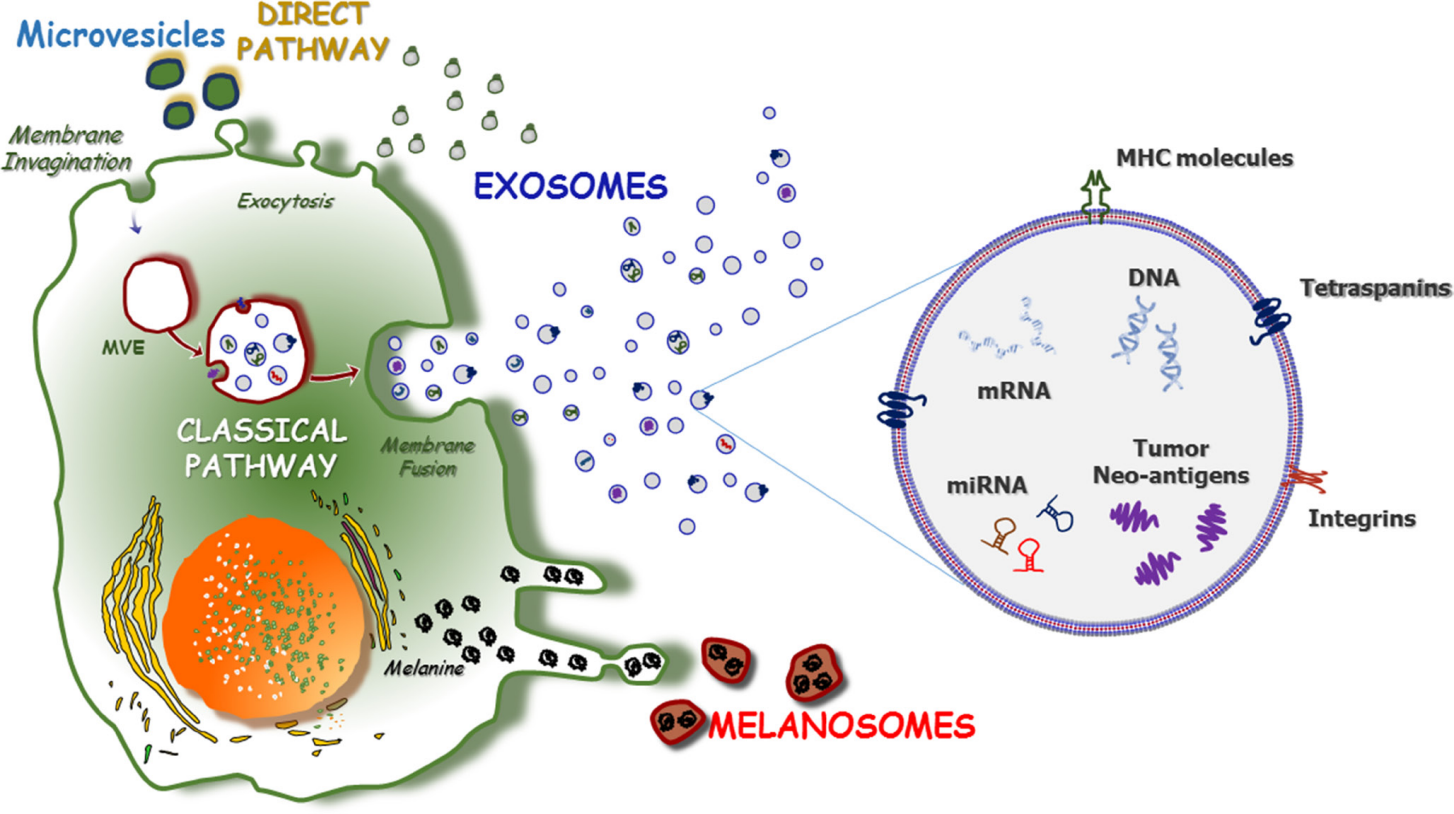
The pathways of exosome formation Two major pathways of Exo formation have been described as ‘direct’ and ‘classical’ (left). The ‘direct pathway’ promotes the Exo formation through the direct exocytosis of vesicles, including nanovesicles and MV stemmed from the outward budding of the plasma membrane. Contrariwise, the ‘classical pathway’ requires the re-activation of endosomes that originates from the inward budding of plasma membrane and leads to the development of multi-vesicular endosomes (MVE). After the active packaging of their content, MVE fuse with the plasma membrane and release Exo in the extracellular space. Exosomes consist of a lipid bilayer (right) that contains trans-membrane and cytoplasmic proteins or non-coding miRNAs, mRNA as well as single-stranded and double-stranded DNAs. Also, exosomes are engulfed of a number of proteins that are mostly represented by tumor-derived neo-antigens, class-I and -II MHC molecules and tetraspanins. Furthermore, the early phases of melanomagenesis are characterized by the production of melanosomes, melanin-containing organelles that result highly enriched of oncogenic miRNAs, whose major role concerns the preparation of the primary metastatic niche milieu.

The formation of Exo and their release into biological fluids occur via two major pathways: classical and direct (Figure 1). The classical pathway involves the development of intraluminal vesicles within multivesicular endosomes (MVE) that fuse either with lysosomes associated with cargo degradation or with the plasma membrane, thus leading to the release of intraluminal vesicles, namely Exo. However, cellular cargo may also interact with target cells through both ligand/receptor activities and the endocytosis of Exo [25, 30]. The trafficking of MVE throughout the membrane or toward lysosomes is regulated by a small GTPase originating from the Rab family [31], while calcium levels and the citron kinase control the final fusion of MVE with the plasma membrane [32]. Moreover, accumulating evidence suggests that endosomal sorting complexes required for transport (ESCRT) play important roles during membrane invagination [33, 34] since they deform the endosomal-limiting membrane by specific protein-protein and protein-lipid interactions. The result is the inward budding of vesicles, with subsequent cargo recognition and sorting by ubiquitin-interacting modules. The ubiquitinated cargo first binds hepatocyte-growth-factor-regulated kinase substrate, a component of ESCRT located on the endosomal membrane; it is then loaded onto the membrane through ESCRT-dependent proteins before it is packaged into budding Exo [35]. The release of Exo can also proceed via an ESCRT-independent route that mostly involves the ceramide pathway [36]. The peculiar properties of ceramide favour vesicle biogenesis, including the cone-shaped structure that promotes a spontaneous negative curvature in the membrane bilayer of Exo [37]. The ceramide-dependent biogenesis of Exo also involves tetraspanins, which form oligomers that interact with a variety of cytosolic proteins [38].

Alternatively, the direct pathway is an immediate route leading to Exo formation and it is mostly used by T-cells for the rapid generation of these vesicles from the plasma membrane. The typical antigenic profile of Exo produced by the direct pathway includes the expression of CD63, CD81 and CD82, although Mal-7 expression is apparently specific for T-cell-derived Exo [39].

## EXOSOMES AND MELANOMA PROGRESSION

**Figure 2:**
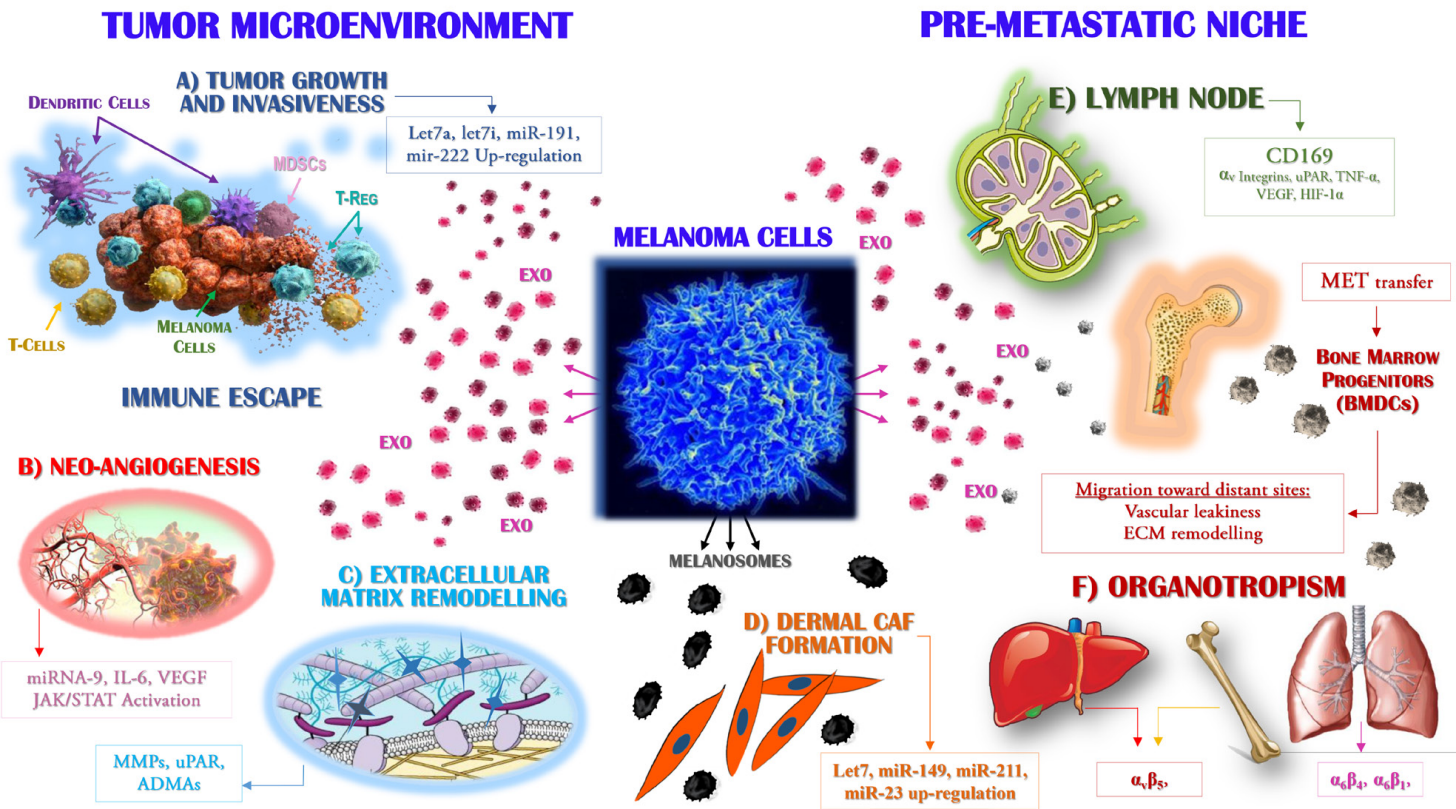
Exosomes drive the metastasis of melanoma cells Primary melanoma cells produce Exo that are able to target distinct populations, both within the tumor microenvironment (left) and at distant sites where they participate to the pre-metastatic niche formation (right). The molecular mechanisms by which Exo interact with target cells include the cytokines, miRNAs and receptors variably expressed by or accumulated in these vesicles. Exo enriched in Let7a, Let7i, miR-191 and miR-222 (**A**) promote tumor growth and invasiveness while are also able to impair immune system activity, leading to the defective maturation of dendritic cells, impaired cytotoxicity of T-cells and the expansion of suppressive populations, including regulatory T-cells (Treg) and myeloid-derived suppressor cells (MDSCs). (**B**) The expression by Exo of miR-9 and the high levels of both interleukin (IL)-6 and vascular endothelial growth factor (VEGF) drive the neo-angiogenesis, through the activation of the JAK/STAT pathway, whereas uPAR and ADAMs proteases (**C**) promote the remodelling and degradation of the extracellular matrix. Melanocytes produce melanosomes (**D**) as vesicles engulfed of melanin that are found accumulated in keratinocytes. Malignant melanoma cells stimulate the collagen-associated fibroblasts (CAFs) of the dermis leading to increased melanoma cell proliferation through the over-production of miR-149, -211, -23, -let7a and -let7b. In addition, the up-regulation of CD169 by Exo is a key step in the recruitment of melanoma cells to lymph nodes (**E**), whose colonisation by metastatic cells is also favoured by α_v_ integrins, hypoxia-inducible factor (HIF)-1α and tumor necrosis factor (TNF)-α. In addition, specific organotropism (**F**) is driven by MET-expressing Exo, which in turn promotes the mobilisation of bone marrow progenitors (BMDCs) implicated in neo-vasculogenesis and pre-metastatic niche formation.

Melanoma develops in a stepwise process involving genetic, epigenetic and environmental factors related to the malignant transformation of melanocytes, allowing their uncontrolled proliferation and their acquisition of invasiveness [3, 40]. The ability of cells to communicate by intercellular contact and/or the secretion of soluble molecules, including growth factors, cytokines and chemokines released by extracellular vesicles, was recently reported [41]. In the case of Exo, these vesicles promote the metastatic spread and hence the progression of melanoma, by enabling cancer cells to escape from immune surveillance and by altering signalling pathways via the transfer of different stimuli that promote angiogenesis and stromal remodelling (Figure 2). Among the major events in melanoma progression are the construction of a vascular network surrounding the tumor bed and/or the maintenance of an immunosuppressive milieu, the interaction between melanoma cells and dermis operated by melanosomes that leads to the formation of dermal tumor niche [42] before tumor dissemination towards distant sites that mostly depends on complex cross-talk between malignant and stromal cells [2, 43].

### Exosomes influence tumor growth, angiogenesis and the invasiveness of melanoma cells

Exosomes influence the pro-metastatic behaviour of melanoma cells (Figure 2A) by altering the intracellular pathways activated by both oncogenes and non-coding RNAs [44]. The over-expression of miRNA-222 in melanoma cells drives tumorigenesis by inhibiting p27, CDKN1B and c-Fos gene expression [45]. This capability can be efficiently transferred by Exo to recipient cells, resulting in the activation of the PI3K/AKT pathway required for enhancing melanoma cell proliferation [46]. Exo also promote neo-angiogenesis and the ability of cancer cells to invade proximal and distant tissues; these activities seem to be regulated by the immuno-modulating agents and pro-angiogenic molecules carried by Exo (Figure 2B), such as interleukin (IL)-6, vascular endothelial factor (VEGF) and particularly MMPs [47]. The remodelling of the ECM by MMPs is essential for tumor cell invasion and metastasis because focal degradation of the ECM is the first step in the invasion of cancer cells. Furthermore, Exo from several tumor models, including melanoma, were shown to be enriched in proteases such as uPAR, ADAMs and HAdase, which mediate the digestion of type I and IV collagens, laminins and fibronectin (Figure 2C). Moreover, Exo highly express CD44 and α_6_β_4_ molecules, which interact with hyaluronic acid and laminin and thereby adhere to extracellular stromal components [48]. The protruding adhesive structures resulting from such interactions have proteolytic activity and by adhering to the ECM dissolve its collagen, laminin and fibronectin components. In addition, invadopodia are key docking sites for Exo strongly impaired by miRNAs produced by tumor cells and targeting Rab27 [49].

Exo also exhibit pro-angiogenic activity, by transferring miR-9 from melanoma to endothelial cells, which triggers the JAK-STAT pathway and enhances the migratory propensity of vascular cells as well as the formation of a tumor-supporting vascular grid [50]. The metastatic propensity of melanoma is also increased based on the ability of the tumor cells to cross either the blood or the lymphatic vessels by rearrangement of their cytoskeleton and by altering their contacts with the ECM [51]. This process, referred to as the EMT, is influenced by Exo, which facilitate the switch such that the mesenchymal rather than the epithelial features of the cells are manifested [52]. Melanoma-derived Exo also promote the EMT by up-regulating Let7a, Let7i and miR-191, which in turn activate the MAPK pathway by down-regulating E-cadherin and by the over-expression of mesenchymal molecules, including vimentin, ZEB2 and SNAIL2 (Figure 2A) [53].

### Melanosomes and exosomes influence the pre-metastatic niche formation and organotropism of melanoma metastases

Melanocytes produce the pigment melanin that is stored in melanosomes (Figure 2D). They are highly specialized organelles engulfed of low (immature) and high (mature) levels of melanin that in normal skin are transferred nearby keratinocytes in response to ultraviolet exposure. Melanosomes control the communication between melanoma cells and microenvironment promoting the formation of cancer-associated fibroblasts (CAFs) that result enriched of genes driving the cell proliferation, motility and inflammation. The mechanisms used by melanosomes for the activation of CAFs include the abnormal production of miRNAs (-149, -211, -23, -let7a and -let7b) that activate the ERB cascade upstream the MAPK signalling contrariwise to Exo that uniquely stimulates the WNT pathway. Based on these findings, it is conceivable that melanosomes establish an early niche within the dermis and contribute to the metastatic phenotype of invasive melanoma cells [42]. Furthermore it has been demonstrated that Exo play a critical pro-tumorigenic effect in the tumor microenvironment [54] while those from melanoma cells also migrate toward distant tissues to prepare the pre-metastatic niche. Previous studies have identified Exo within sentinel lymph nodes, where their preparation of a favourable niche for melanoma cell homing and growth was demonstrated [55]. Exo recruited into lymph nodes up-regulate the proteases that degrade the ECM as well as the pro-angiogenic factors tumor necrosis factor (TNF)-α, VEGF, hypoxia-inducible factor (HIF)-1 and urokinase plasminogen activator, thus enhancing melanoma cell recruitment, trapping and growth (Figure 2E) within the metastatic niche [56, 57]. Exo may also prime bone-marrow-derived cells (BMDCs) to acquire a vasculogenic, metastatic phenotype, in addition to driving the horizontal transfer of the oncoprotein receptor tyrosine kinase MET, which enhances the mobilisation of BMDCs and thus facilitates their recruitment to metastatic sites [58]. Therefore, BMDCs enhance the propensity and extent of metastatic disease by establishing a suitable microenvironment for trapping circulating melanoma cells [54].

In the progression of melanoma, Exo directly influence organotropism (Figure 2F). The fusion of these vesicles with their target cells prepares the recipient tissue such that it becomes permissive for the subsequent homing of metastatic cells. Proteomic analyses of tumoral Exo revealed that their distinctive patterns of integrin expression correlated with specific behaviours, with the up-regulation of α_6_β_4_ and α_6_β_1_ mostly leading to lung tropism, and that of α_v_β_5_ hepatic tropism [59]. Other studies demonstrated that the MET oncoprotein accumulates in Exo-derived from melanoma cells with a selective tropism for the lung [60] whereas the fusion of Exo with fibroblasts up-regulates Src and S100, which in turn stimulate both chemotaxis and inflammation [59].

## MELANOMA EXOSOMES AND THE IMMUNE SYSTEM

**Figure 3:**
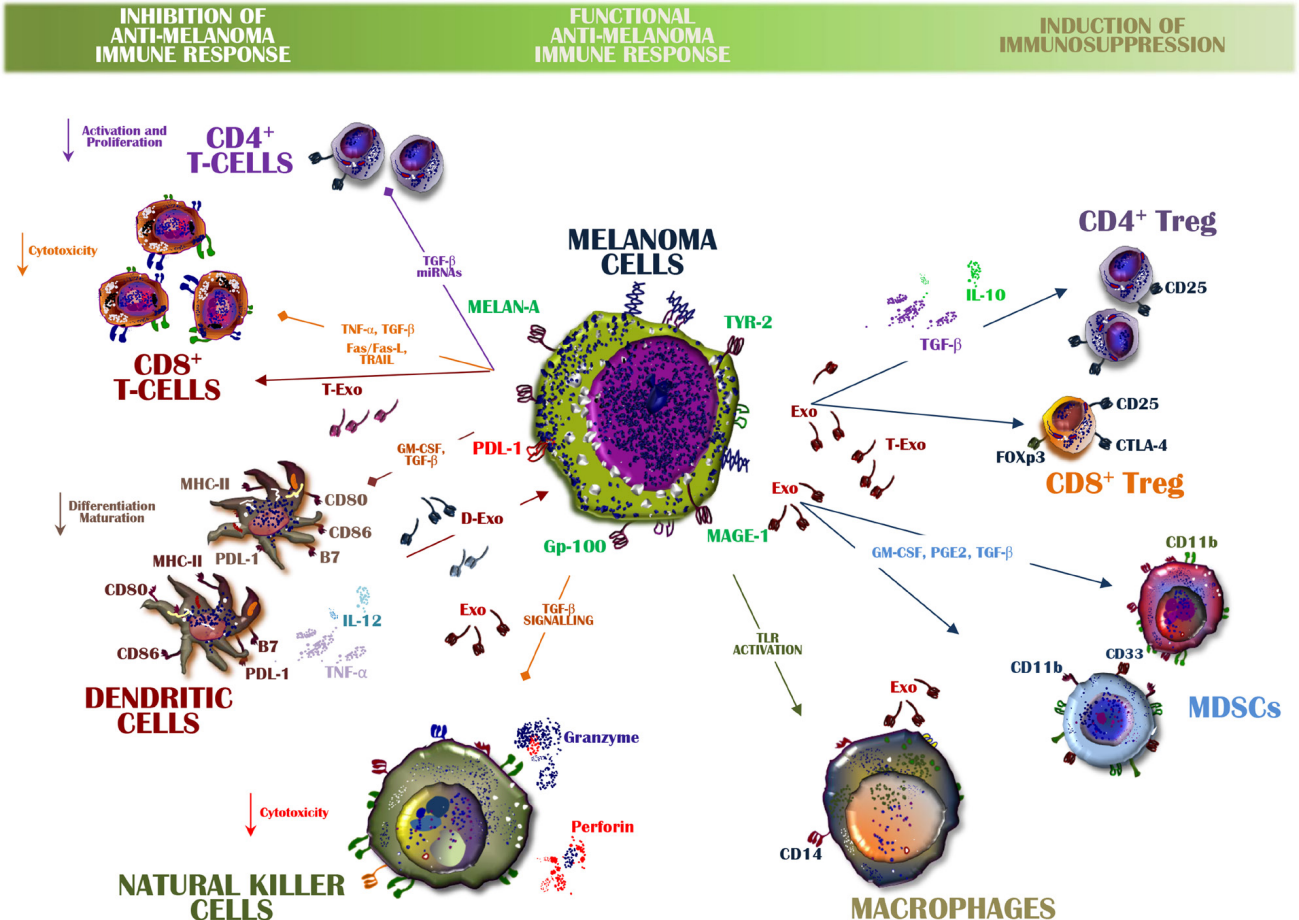
Exosomes from melanoma cells balance immune system activity A functional anti-melanoma immune response is orchestrated by Exo containing inhibitory and stimulatory molecules that may alter the immune system balance. The expansion of Tregs and MDSCs is also induced by up-regulated transforming growth factor (TGF)-β or by inhibitory signals directed from melanoma-derived Exo toward both CD8^+^ T-cells and DCs. Among the immune-suppressive cytokines involved in these processes are interleukin (IL)-6 and IL-10 as well as pro-apoptotic molecules, all of which may be transferred from Exo to immune cells. Conversely, exosomal TGF-β and toll-like receptor (TLR) activation impair the release of perforins and granzyme by NK cells as well as macrophage activation. However, also immune cells produce Exo within the microenvironment and those from DCs (D-Exo) and CD8^+^ T-cells (T-Exo) interplay with both tumor cells and immune cells leading to melanoma evasion from the immune system control.

Immune editing depends upon complex machinery that includes intra- and extracellular signals aimed at counterattacking both proliferation of malignant cells and tumor progression. Its deregulation is therefore critical for the escape of melanoma cells from immune system control [2, 61]. Exo play a role in immune escape, both directly and indirectly (Figure 3). The direct modulation of either immune cells or their immature precursors largely occurs in response to Exo-related inhibitory or pro-apoptotic signals during the migration of melanoma cells to distant tissues. The indirect role of Exo involves the expansion and differentiation of negative regulators of the immune system, such as MDSCs and Tregs, thus favouring tumor cell escape from immune surveillance [62, 63].

Recent observations from immune-based cancer therapies have provided support for a strategy based on the harnessing of the cytotoxicity of CD8^+^ T-cells to destroy tumor cells [64–66]. Immunotherapy sustains and enhances the efficiency of T-cell response, but this T-cell population is subject to tolerance-inducing mechanisms most of which are activated in the tumor microenvironment and ultimately limit the effectiveness of these immune cells. Tumor-derived Exo are enriched in the immune inhibitors produced by melanoma cells and by tolerogenic immune cells; these molecules hamper both immune recognition and the elimination of melanoma cells, with the latter subsequently acquiring a proliferative capability. Among the Exo-related mechanisms that alter immune system activity are the stimulation of selective ligand/receptor interactions in T-cells or their internalization through phagocytosis by DCs [67]. Treg are particularly sensitive to Exo, whereas CD4^+^ T-cells are mostly inhibited by these vesicles once the adenosine pathway becomes activated [68]. Therefore, Exo from melanoma cells are able to interfere with the majority of immune cells and thereby enhance the signals that allow the propagation of tumor cells to distant tissues.

Moreover, the melanoma microenvironment also contains Exo produced by either immune or stromal cells and thus involved in the inhibition of melanoma cell proliferation. These observations suggested the use of Exo for therapeutic purposes; indeed, a number of vaccination trials based on Exo derived from DCs and NK cells are currently in progress in clinical settings [19, 69]. Also immune cells, indeed, release Exo as DCs, mast cells as well as B- and T-cells [70, 71]. In this context, it has been shown that Exo from human and experimental B-cells induce an antigen-specific MHC class-II restricted T cell responses, thus suggesting their primary role in the antigen presentation [72]. Moreover, T-cell-derived Exo are key mediators of inflammation and immune response as well as modulate the activity of immune cells and dampen the cytotoxicity of auto-reactive T-cells, thus restraining transplant rejection [73, 74]. Lastly, DCs release MVs named dexosomes (Dex) that result enriched of CD80, CD86 co-stimulatory molecules for the functional antigen presentation required for the amplification of the immune response and the modulation of the anti-tumor activity in cancer [75].

### Effector T cells (Teffs)

Exosomes can inhibit T-cell activity by delivering membrane-bound ligands to the cognate receptors expressed on immune cells. The transfer and delivery of information from Exo to immune cells are critical elements of the cross-talk that occurs between tumor cells and the host immune system. In fact, many of the signals delivered by Exo do not require internalisation and may change both the phenotype and the function of recipient T-cells, resulting in an impairment of the cytotoxic immune response. In addition, the TCR-CD3 *ζ*-chain complex, necessary for the immune response to foreign antigens, is negatively influenced by melanoma Exo, as are JAK3 and STAT5, whose activation and phosphorylation up-regulate lymphocyte proliferation [62]. Exo also trigger apoptosis in T-cells, both through the Fas and TRAIL death domains and the activation of the PD1/PDL-1 pathway, while expressing a number of immune suppressive molecules, such as transforming growth factor (TGF)-*β*1 and several negative regulators of T-cell cytotoxicity, including IL-10 and CTLA-4 [76–78].

However, in contrast to their prevalent immune inhibitory effects, Exo are also able to promote a robust anti-melanoma response by T-cells. This ability is favoured by the high-level expression by Exo of MHC class I molecules and other antigens (gp100, TYR2 and MART1). These molecule interact with T-cells to potentiates their cytotoxic anti-melanoma activities [79]. Recent studies of the intercellular cross-talk in melanoma demonstrated that the Exo payload is shaped by somatic evolution, as Exo from different melanoma cell lines exert variable effects on the T-cell-inhibited proliferation of the tumor cells, including via the exosomal up-regulation of the protein-tyrosine phosphatase PTPN11 [27].

### T regulatory cells

The mechanisms responsible for the immune escape of tumor cells include expansion of the Treg population in circulating blood as well as within the primary tumor and at metastatic sites. However, while Exo interact with many T-cell subtypes, their major effect is on the inhibitory population that over-produces IL-10 and TGF-β1, increases the expression of negative immune checkpoints such as CTLA-4 and hyper-phosphorylates SMAD2/3, which has been implicated in the transcription of intracellular inhibitory signals that restrain the anti-melanoma immune response [62]. In other experimental models of cancer, the transfer of miRNA-214 from tumor-derived Exo to T-cells down-regulates PTEN (phosphatase and tensin homolog) while favouring the expansion and migration of Treg in the cancer microenvironment [80]. Moreover, Exo induce tolerogenic DCs, which also enhances Treg generation. These observations are supported by the finding that the T-cells in a B16 melanoma model are poorly cytotoxic, as a result of the negative signals driven by Treg-derived Exo [81]. Finally, Exo modulate the levels of the immune checkpoint proteins of Treg. This property may be relevant in the design of new anti-melanoma immunotherapies.

### Monocytes and dendritic cells

Previous studies demonstrated that melanoma-released vesicles impair monocyte differentiation and maturation of DCs. The monocytes associated with melanoma are restrained in their expression of CD80 and CD86 co-stimulatory molecules but retain CD14 and HLA class II expression, thus finally promoting the expansion of CD11b^+^/Gr-1^+^ MDSCs [82]. Therefore, monocytes from melanoma progressively acquire a CD14^+^/HLA-DR^-/low^ suppressive phenotype that alters T-cell proliferation and interferon-γ production while enhancing the secretion of inhibitory cytokines such as IL-6, TNF-α and TGF-β within the tumor milieu [83–85]. Additional studies in IL-6 knockout, melanoma-bearing mice demonstrated that a high content of this cytokine in tumor Exo inhibits DC differentiation from monocyte precursors, via a mechanism that includes the transcription of kinases upstream and downstream of STAT-3, while inducing tolerance and therefore the escape of melanoma cells from immune system control [14]. In contrast to the prevalent inhibitory effect of melanoma Exo on DC activity, a study using the B16_F1_ melanoma cell line showed that these vesicles may alternatively induce DC maturation and thus the proliferation of T-cells [86]. The latter finding suggests a therapeutic application for Exo. In other studies, antigens exposed by tumor Exo could be transferred to DCs, where they activated cytotoxic T-cells, although the latter exhibited only a weak anti-cancer immune response. In line with these findings, Dex from circulating DCs apparently exert a potent anti-melanoma activity in terms of antigen presentation and the cross-priming of CD4^+^ and CD8^+^ T-cells as well as an ability to strengthen both adaptive and innate immunity. Dex vaccination was therefore tested in a phase I clinical trial in patients suffering from advanced melanoma but the improvement in survival was minimal. Further efforts are needed to potentiate the clinical efficacy of Dex *in vivo* [19].

### Natural killer cells

A specific role of melanoma-derived Exo on NK cell activity has not been described, although in other cancer models the respective Exo apparently interfere with a number of receptors implicated in NK-driven cytotoxicity. In particular, cancer-derived Exo were shown to restrain NKG2D, NKp30, NKP46 and NKG2C receptor expression via the up-regulation of TGF-β and the phosphorylation of SMAD, resulting in the inhibition of granzyme and perforin release, required for the cytotoxic effect of NK cells on malignant cells [76]. However, NK cells themselves release Exo, as demonstrated in the NK-92 cell line, which releases high levels of CD63, ALIX and Fas-L, all of which participate in death-domain mediated apoptosis. As these Exo are able to induce anti-melanoma activity both *in vitro* and *in vivo*, their use as an immune-therapeutic strategy in metastatic melanoma has been proposed [87].

## CLINICAL AND TRANSLATIONAL USE OF EXOSOMES IN MELANOMA

The identification of predictive and prognostic biomarkers in cancer is an active area of research, but thus far only a few such molecules are relevant for clinical application [88, 89]. However, several recent studies have proposed the use of Exo in cancer, for diagnostic and prognostic purposes. Exo isolated from the sera of melanoma patients express higher amounts of CAV-1, S100B, and MIA (melanoma inhibitory activity) than measured in healthy controls. Further studies of the potential prognostic role of Exo in melanoma demonstrated a negative correlation between high levels of CAV-1^+^ Exo and outcome [23, 90]. These studies have motivated further investigations in other cancer models, including prostate cancer, given that prostate-specific antigen levels are not a reliable indicator of either the early phases or the recurrence of prostate cancer, whereas the prostasomes released by prostate cancer cells may act as a biomarker. Indeed, prostasome enrichment in the circulation and urine of prostate cancer patients apparently correlates with the extent and course of the disease. Similar results have been obtained in ovarian cancer and glioblastoma and in the immune cells of metastatic melanoma patients. In the latter, the antigen profiles of Exo from T-cells (T-Exo) and Dex are very similar to those of the parent cells, including the expression of putative immune checkpoint receptors, which can be clinically targeted by immunotherapy, and co-stimulatory molecules such as CD80 and CD86. Moreover, high-level expression of PD1 and CD28 by T-Exo is indicative of a clinical benefit from ipilimumab and correlates significantly with progression-free and overall survival. Therefore, PD1 and CD28 expression by T-Exo may be a valuable tool in predicting the best responders to immunotherapy among patients with melanoma, while CD80 and CD86 levels may serve as prognostic biomarkers [24].

Besides the antigenic repertoire exhibited by Exo [91], these vesicles transport high amounts of RNA and DNA, a feature of diagnostic and prognostic relevance. Increased levels of miR-17, miR-19a, miR-21, miR-126, and miR-149 have been measured in Exo from metastatic melanoma patients and their use in the monitoring of clinical outcome has been described [92]. Similarly, the double-stranded DNA of Exo may be useful for the detection of BRAF mutations [93, 94]. In addition, high levels of miR-211 in melanoma-derived Exo were shown to indicate a reduced sensitivity to BRAF inhibitors, the mechanism of which involves the over-expression of MITF, a regulator of the TRPM1 gene, resulting in the prolonged survival of melanoma cells [95]. Other clinical applications include the use of Exo to deliver drugs or miRNAs to tumor cells or to stimulate the immune response by exploiting the antigenic repertoire of the vesicles. Of relevance to the latter is the finding that Exo from tumor-peptide-pulsed DCs may prime specific cytotoxic lymphocytes and thereby suppress cancer growth in a T-cell dependent manner [70]. A phase I clinical trial of Exo-based vaccination demonstrated the feasibility of large-scale Exo production and the safety of Exo administration [19].

Notwithstanding early studies were completed in small groups of patients limiting the clinical impact of achieved results, tumor-derived Exo appear a very interesting tool for the characterization of the molecular signatures of melanoma. Therefore, future clinical trials might address their diagnostic and predictive role in clinical practice while the therapeutic application still appears unripe for forthcoming clinical applicability.

## CONCLUSIONS

Exosomes exert a wide range of biological functions, primarily via cell-to-cell cross-talk and the delivery of effectors or signalling molecules that regulate diverse cellular processes. Because they also contribute to cancer development and metastasis, their detection in a variety of biological fluids represents a promising strategy to gain pathogenic information and to identify specific biomarkers of diagnostic and prognostic relevance. In the era of precision medicine, the great promise of Exo concerns their potential application to non-invasive strategy aimed at the early definition of biomarkers to identify the responders to immunotherapy or for discovering specific dysfunctions of the immune system implicated in the melanoma development and spreading to distant tissues. Moreover, Exo may be useful in predicting the therapeutic response and, in modified form, in the targeting of specific organs via the systemic administration of miRNAs, siRNAs, and chemotherapy. In this context, these enriched Exo might be ideally used as stimulating adjuvants against neo-antigens released during the tumor shrinkage during immunotherapic strategies. While, thus far, very little is known about the biology of Exo, they are currently a topic of active research. Those findings will improve our understanding of the role of Exo in cancer development and progression.
